# Phosphatidylinositol-4-phosphate signaling regulates dense granule biogenesis and maturation in *Toxoplasma gondii*

**DOI:** 10.1371/journal.ppat.1014451

**Published:** 2026-07-24

**Authors:** Angela Arabiotorre, Megan Formanowicz, Vytas A. Bankaitis, Aby Grabon

**Affiliations:** 1 Department of Cell Biology & Genetics, College of Medicine, Texas A&M Health Sciences Center, College Station, Texas, United States of America; 2 Department of Chemistry, Texas A&M University, College Station, Texas, United States of America; UTSW: The University of Texas Southwestern Medical Center, UNITED STATES OF AMERICA

## Abstract

Phosphoinositide metabolism defines the foundation of a major signaling pathway that is conserved throughout the eukaryotic kingdom. Although the 4-OH phosphorylated phosphoinositides phosphatidylinositol-4-phosphate (PtdIns4P) and phosphatidylinositol-4,5-bisphosphate are each individually required for the viability of all eukaryotic cells studied thus far, their activities in parasite biology are less well understood. Using intracellular tachyzoites of the apicomplexan parasite *Toxoplasma gondii* as model for studying PtdIns4P signaling in a protozoan, we demonstrate the presence of PtdIns4P pools in Golgi/trans-Golgi (TGN) system and in post-TGN compartments of the parasite. Moreover, using a combination of super-resolution confocal microscopy and correlative light electron microscopy, we show that deficits in PtdIns4P signaling result in structural perturbation of compartments that house dense granule cargo with accompanying deficits in dense granule exocytosis. Taken together, the data report a direct role for PtdIns4P in dense granule biogenesis and exocytosis. The data further suggest that the biogenic pathway for secretion-competent dense granule formation in *T. gondii* is more complex than simple budding of fully matured dense granules from the TGN.

## Author summary

*Toxoplasma gondii* is an obligate intracellular parasite that infects most species of warm-blooded animals. To execute its parasitic life cycle, *T. gondii* utilizes a set of specialized late secretory organelles – micronemes, rhoptries, and dense granules – to secrete effector proteins that allow the parasite to execute successful invasion, intracellular survival, and egress from host cells. While some aspects of microneme and rhoptry biogenesis have been described, comparatively little is known regarding dense granule biogenesis. Current models envision a simple and direct mechanism of dense granule biogenesis in *T. gondii* as intermediate maturation steps in this process have not been observed. In this work, we arrest dense granules (DGs) in what we interpret is an immature form by interfering with phosphatidylinositol-4-phosphate (PtdIns4P) signaling. We interpret these results to suggest that DGs undergo a multi-step PtdIns4P-dependent maturation process necessary for efficient cargo secretion. Whereas PtdIns4P in yeast and mammalian cells is an essential potentiator of exocytic trafficking from the TGN to the plasma membrane, the roles of PtdIns4P signaling in *T. gondii* have not been investigated. This report is the first to address the roles of PtdIns4P signaling in *T. gondii* and identify PtdIns4P signaling in promoting TGN/post-TGN membrane trafficking steps from the TGN as a primordial function for this phosphoinositide.

## Introduction

Phosphoinositides (PIPs) are phosphorylated derivatives of the glycerol-based phospholipid phosphatidylinositol (PtdIns). PIPs are chemically distinguished by positionally specific phosphorylations at the 3-OH, 4-OH and 5-OH positions on the inositol ring that constitutes the headgroup of these lipids [1]. The relatively low chemical complexity of the PIP cohort notwithstanding (mammals express seven PIPs while yeast produce four) these molecules regulate literally hundreds of distinct biological outcomes [reviewed in 2–4]. Among the various cellular events regulated by PIPs, maintenance of organelle identity and regulation of membrane trafficking events is of direct relevance to this study. In yeast and higher eukaryotes, PIP-regulated membrane trafficking processes include: (1) endocytosis and the formation of PtdIns(3)P-positive vesicles from the plasma membrane (PM) and its fusion with the endocytic system [5–9], (2) the PtdIns(3)P-dependent formation of autophagosomes and PtdIns(3,5)P_2_-dependent fusion with lysosomes [10], and (3) formation of exocytic vesicles at the TGN in a PtdIns4P-dependent manner and their subsequent transport to the plasma membrane [11–14]. Considerable effort has been invested in the identification and characterization of protein factors that function as downstream effectors of PIP signaling in these various membrane trafficking events – primarily in yeast and in mammalian cells [15–17]. Little is known about the relationship between PIP signaling and membrane trafficking in protozoans, however [15–17].

Organisms of the phylum Apicomplexa are complex single-celled eukaryotes that exhibit an obligate intracellular parasitic lifestyle that comes with unique adaptations. Apicomplexans evolved from the secondary endosymbiosis of a photosynthetic alga by an ancestral eukaryotic cell [18,19]. As a result, these species offer an ideal opportunity to investigate the physiological integration of PIP signaling systems in unicellular cells that followed evolutionary trajectories that led to parasitism. In the case of Apicomplexa these trajectories represent the transitions from free-living phototrophs to obligate non-photosynthetic animal parasites. *T. gondii* is arguably the most experimentally tractable apicomplexan parasite. This organism can be cultured in vitro, is amenable to genetic manipulation, and rodent models have been established for studying *T. gondii* infection [20,21]. Like other apicomplexans, *T. gondii* harbors unique endomembrane organelles dedicated to the execution of activities essential for completion of their parasitic life cycles. A set of specialized late secretory organelles – micronemes, rhoptries, and dense granules (DGs) – store and secrete the cargos required for invasion, intracellular survival, and egress from host cells [22–25]. Although some factors involved in the biogenesis and function of micronemes and rhoptries have been characterized, the roles of PIPs in secretory organelles in *T.gondii* remain largely unknown.

PIP binding domains have been successfully used to visualize intracellular distributions of PtdIns3P and PtdIns(3,5)P_2_ in *T. gondii*, and to reveal 3-OH PIPs involvements in the biogenesis and/or inheritance of the apicoplast [26; 27]. The report of a Golgi pool of PtdIns4P in *Plasmodium falciparum* notwithstanding [28], the biological function of PdIns4P has not been studied in detail in either *T. gondii* or in Apicomplexa [29]. Herein, we provide functional information regarding PtdIns4P signaling in intracellular tachyzoites of *T. gondii*. Our data demonstrate the presence of PtdIns4P pools in the Golgi/TGN system and post-TGN compartments and provide evidence for cell-cycle-dependent regulation of PtdIns4P signaling. Moreover, we show that compromised PtdIns4P signaling results in structural perturbation of DGs with accompanying deficits in exocytosis of DG cargo. Taken together, the data demonstrate that PtdIns4P itself is a key regulator of DG biogenesis/maturation and exocytosis and indicate that the biogenic pathway for DG formation in *T. gondii* is more complex than presently thought [30].

## Results

### Visualization of *T. gondii* PtdIns4P pools by genetically encoded biosensors

To visualize pools of PtdIns4P and its higher-order derivatives PtdIns(4,5)P_2_ and PtdIns(3,4)P_2_ in *T. gondii*, fluorescent biosensors based on PIP binding domains of known specificity were expressed in the parasite and imaged. A tandem human PLCδ PH domain fused to enhanced green fluorescent protein at the C-terminus (2xPHPLCδ-EGFP; [31]) and a PH domain of human TAPP1 fused to YFP at the N-terminus (YFP-TAPP1PH; [32]) were used to monitor distribution of PtdIns(4,5)P_2_ and its regioisomer PtdIns(3,4)P_2_, respectively. Expression of these biosensors was driven by the tubulin (Tub1) promoter. The corresponding reporter genes were transfected into *T. gondii* RH tachyzoites and their profiles recorded under transient expression conditions. Parasites were allowed to infect a monolayer of human foreskin fibroblasts (HFF-1) cells and wide-field fluorescence imaging performed 24 hours post-transfection (hpt) reported recruitment of both biosensors to the plasma membrane of intracellular parasites (Fig 1A). These data suggest both PIP_2_ regioisomers are predominantly localized to the plasma membrane of *T. gondii*. We note that substantial cytoplasmic pools were also detected for both biosensors -- indicating plasma membrane recruitment of these PIP_2_ biosensors is less efficient in *Toxoplasma* than in other eukaryotes [33–35].

**Fig 1:**
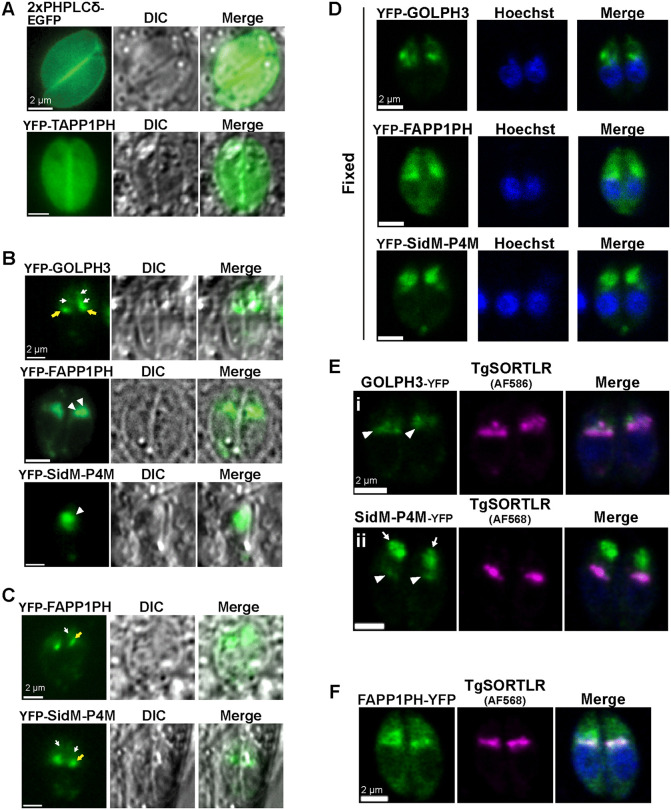
Imaging of PtdIns(4,5)P_2_ and PtdIns(3,4)P_2_ at the plasma membrane and PtdIns4P pools at Golgi/TGN and post-TGN compartments using specific biosensors. **(A-B)** Widefield fluorescence microscopy of live intracellular parasites expressing the indicated PIP biosensors are shown. **(A)** PtdIns(4,5)P_2_ (2xPHPLCδ-EGFP) and PtdIns(3,4)P_2_ (YFP-TAPP1PH) localize to the plasma membrane. **(B)** PtdIns4P pools in RH-GOLPH3 cells localize to a single stack-like structure (yellow arrows) and scattered anterior small vesicles (white arrows); PtdIns4P pools in RH-FAPP1PH and RH-SidM-P4M strains localize to the LAP body (arrowheads). **(C)** RH-FAPP1PH and RH-SidM-P4M cells expressing low levels of the respective biosensors show PtdIns4P in a single stack-like structure (yellow arrows). **(D)** Fluorescence images of biosensor localization in RH-GOLPH3, RH-FAPP1PH and RH-SidM-P4M strains. In RH-GOLPH3 and RH-SidM-P4M parasites. The localization of the biosensor is conserved following fixation, while RH-FAPP1PH cells show a more diffused pattern when compared to the profiles seen in live cells. Parasite DNA is visualized using DAPI (blue). **(E-F)** Confocal images of immunofluorescence analysis (IFA) for the identification of Golgi/TGN (using anti-TgSORTLR immunoglobulin) in parasites during G1/S phase. **(E)** GOLPH3-YFP and SidM-P4M-YFP colocalize with TgSORTLR (arrowheads). The LAP body forms in a post-Golgi/TGN compartment in RH-SidM-P4M cells (arrows), but not in RH-GOLPH3 cells. **(F)** The LAP body forms in a post-Golgi/TGN compartment in RH-FAPP1PH cells.

PtdIns4P is a metabolic precursor for both PtdIns(4,5)P_2_ and PtdIns(3,4)P_2_ and serves essential signaling functions in all eukaryotic cells. To monitor PtdIns4P distribution in *T. gondii*, three independent biosensors were designed that exploit distinct PtdIns4P-specific binding modules with differing binding affinities. These included in order of increasing affinity: (i) the N-terminus of human GOLPH3 (K_D_ = 2.6 ± 0.2 μM), (ii) the PH domain of human FAPP1 that we refer to as FAPP1PH (K_D_ = 200 nM at pH 6.5 and 460 nM at pH 7.4), and (iii) the P4M domain of the *Legionella pneumophilia* SidM protein (K_D_ = 3.8 ± 2.7 nM) [36–39]. These reporters detect distinct, but overlapping, pools of PtdIns4P in secretory pathway organelles of mammalian cells and yeast [40–44] (S1A Fig). The transfection and imaging regime for these experiments was as described above for the PtdIns(4,5)P_2_ and PtdIns(3,4)P_2_ biosensors.

In reporting the results, all transiently transfected RH-WT parasites are heretofore referred to by the convention of RH followed by the designation of the corresponding PtdIns4P-binding domain -- i.e., RH-GOLPH3, RH-FAPP1PH and RH-SidM-P4M. Unless otherwise stated, all cells analyzed were not undergoing cell division and are broadly described as G1/S cells. Wide-field fluorescence imaging reported multiple PtdIns4P distribution profiles in G1/S cells as a function of the specific biosensor used to image PtdIns4P profiles (Fig 1B). For example, the biosensor localized to a single juxta-nuclear stack and to apically distributed punctate structures in RH-GOLPH3 parasites. These apical structures appeared as single large and intensely stained puncta in both RH-FAPP1PH and RH-SidM-P4M parasites -- especially in RH-SidM-P4M parasites (Fig 1B, arrowheads). We refer to these apical structures as LAP bodies (Large Anterior Punctate body).

The appearance of LAP bodies was induced by expression of high affinity PtdIns4P biosensors as evidenced by the following observations (S1B Fig). First, expression of the higher affinity FAPP1PH- and SidM-P4M-based biosensors induced LAP body formation, whereas expression of the lower affinity GOLPH3-based biosensor did not. Second, parasites with lower FAPP1PH and SidM-P4M reporter expression (as judged by fluorescence intensity) showed biosensor distributions that recapitulated those of GOLPH3 biosensor-expressing parasites (Fig 1C).

### Multiplicity of PtdIns4P pools

The identities of PtdIns4P-containing organelles were determined in double-label immunofluorescence analyses using antibodies directed against established markers for specific parasite compartments. In these efforts, we first assessed whether the localization of PtdIns4P biosensors in parasites fixed with 4% paraformaldehyde (PFA) recapitulated the profiles observed in live cells. Indeed, the localization profiles of GOLPH3 and SidM-P4M domains in fixed parasites were consistent with those observed in live cells (Fig 1D). However, while the juxta-nuclear compartment localization and LAP body were both detected in fixed RH-FAPP1PH parasites, a diffuse cytosolic signal was also observed (Fig 1D). We interpret that cytosolic signal as a fixation artifact that had previously been observed in FAPP1PH imaging experiments with mammalian cells [45]. Therefore, further use of the RH-FAPP1PH was restricted to live parasite experiments whereas RH-SidM-P4M was deployed in immunofluorescence studies using fixed parasites.

As significant pools of PtdIns4P are present in Golgi/trans-Golgi network (TGN) membranes in yeast and mammalian cells [11,12,46], immunofluorescence approaches were used to assess RH-GOLPH3 and RH-SidM-P4M co-localization with the *T. gondii* TGN marker TgSORTLR (Sortilin-like Receptor) [47]). Both PtdIns4P biosensors colocalized with TgSORTLR to varying degrees. The GOLPH3 biosensor localized to Golgi/TGN membranes (Pearson’s correlation coefficient ± SEM = 0.62 ± 0.03; Fig 1Ei, arrowheads). Recruitment of SidM-P4M to the Golgi/TGN compartment was less efficient but nonetheless detectable (Pearson’s correlation coefficient ± SEM = 0.14 ± 0.04; Fig 1Eii, arrowheads). We interpret this reduced degree of colocalization to be the result of a strong SidM-P4M association with the LAP body (Fig 1Eii, arrows). The FAPP1PH biosensor also targeted to both Golgi/TGN membranes and the LAP body (Fig 1F). Taken together, these data (i) confirm that the stacked juxta-nuclear compartment to which the PtdIns4P biosensors localizes represents the Golgi/TGN system and (ii) suggest that LAP body formation arises from PtdIns4P deficit. The latter issue is the focus of this work.

### The LAP body contains multiple DG cargo

What is the nature of the LAP body to which the SidM-P4M biosensor shows such high affinity? The fact that it does not co-localize with the perinuclear DAPI-positive structure indicates that this is not a perturbed apicoplast. The most attractive possibility was that this structure represented a PtdIns4P-decorated secretory organelle. To examine this possibility in further detail, we assessed co-localization of RH-SidM-P4M with the microneme (marked by MIC3) and the rhoptry neck (marked by RON9) (Fig 2A). As illustrated in Fig 2B, the SidM-P4M biosensor failed to colocalize with either the microneme or the rhoptry markers. Moreover, the morphologies of those secretory organelles were not obviously perturbed in SidM-P4M-expressing parasites when compared to RH-WT cells stained for MIC3 or RON9.

**Fig 2:**
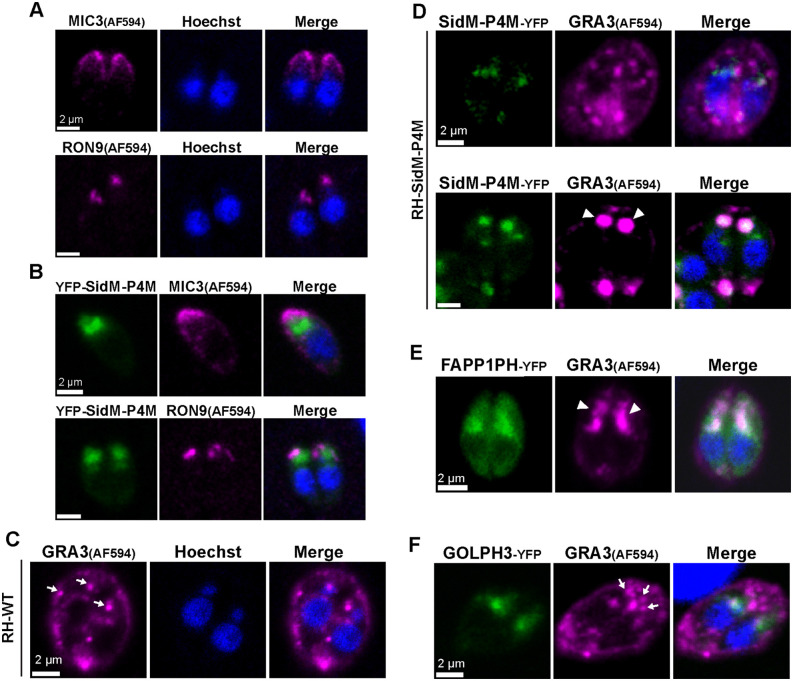
Specific derangement of DG cargo-carrying compartments in parasites expressing high affinity-PtdIns4P biosensors. Confocal microscopy images of fixed RH-WT and transgenic strains are shown. Parasite DNA is visualized using Hoechst (blue). **(A)** Microneme (MIC3) and rhoptry neck (RON9) are all morphologically unperturbed in the parasite. The former two profiles were generated from fixed cells immunostained with the appropriate antibodies. RFP imaging was performed with live cells. **(B)** Distribution of microneme (MIC3), rhoptry neck (RON9) and rhoptry bulb (ROP1) markers is not affected by expression of YFP-SidM-P4M. **(C-F)** Profiles of the DG cargo GRA3 are shown. These were obtained using fixed cells in the G1/S phase of the cell cycle immunostained with anti-GRA3 immunoglobulin. **(C)** RH-WT contain DGs of consistent dimensions dispersed throughout the cytoplasm (arrows). **(D)** RH-SidM-P4M parasites with low and high expression levels of YFP-SidM-P4M are stained with anti-GRA3 antibodies (arrowheads in lower panel show GRA3-positive LAP body). Cells with low expression levels of YFP-SidM-P4M (as reported by fluorescence signal) do not form a LAP body and show WT-like GRA3 distributions (upper panel). **(E)** RH-FAPP1PH parasites show a LAP body stained with anti-GRA3 antibodies (arrow heads). **(F)** DG marker distribution (arrows) is not affected in RH-GOLPH3 parasites.

By contrast, DG profiles were significantly deranged by SidM-P4M expression. RH-WT parasites immunostained for the DG marker GRA3 presented small DG puncta dispersed throughout the cytoplasm (arrows in Fig 2C). Parasites expressing RH-SidM-P4M exhibited two distinct DG profiles that were related to the level of biosensor expression. RH-SidM-P4M parasites with low fluorescence signals (low PtdIns4P biosensor expression) displayed DGs with morphologies and intracellular distributions that were similar to those of WT parasites. Moreover, GRA3 weakly colocalized with the PtdIns4P biosensor (Pearson’s correlation coefficient ± SEM = 0.337 ± 0.013; Fig 2D upper panel). The predominant population of RH-SidM-P4M parasites exhibited more intense fluorescence signals (high level SidM-P4M-YFP expression). Those parasites showed a much stronger co-localization of SidM-P4M-YFP with GRA3 and this colocalization was at the LAP body (Pearson’s correlation coefficient ± SEM = 0.893 ± 0.015; Fig 2D lower panel). Similar results were observed in RH-FAPP1PH parasites (Fig 2E). By contrast, the intracellular distributions and the morphologies of GRA3-positive DGs in RH-GOLPH3 parasites recapitulated those of RH-WT (Fig 2F). However, we did observe rare cases where RH-GOLPH3 parasites exhibited LAP body-associated GRA3 profiles that resembled those of RH-SidM-P4M cells. In those parasites, YFP-GOLPH3 was expressed at particularly high levels.

The immunostaining results indicating a relationship between the LAP body and DGs were further supported by live imaging experiments in cells expressing two genetically-encoded reporters for DG cargo —GRA3-RFP and GRA2-RFP. Those constructs allowed monitoring of DG cargo pools synthesized after parasite transfection and subsequent infection of host cells. RH-WT cells were co-transfected with reporter constructs for each DG cargo and an appropriate PtdIns4P biosensor construct. Live parasites were subsequently imaged. Fifty parasitophorous vacuoles (PVs) were analyzed in three independent biological replicates for each experimental condition, and DG intracellular distribution was scored as: (i) no visible vesicles in the cytoplasm (DG-less), (ii) dispersed vesicles of typical morphology (Normal), or (iii) LAP body containing (LAP body) (Fig 3).

**Fig 3:**
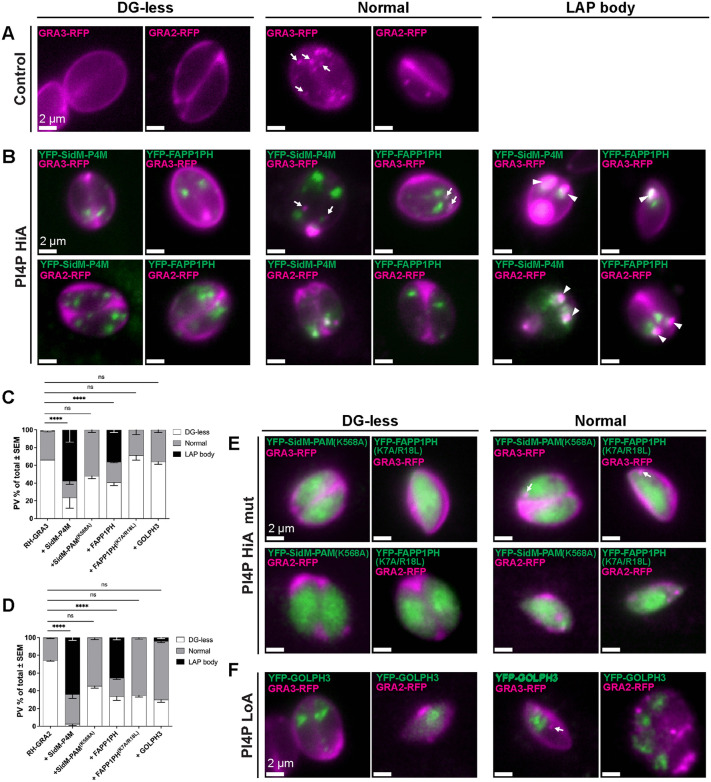
LAP body formation is induced by high affinity-PtdIns4P biosensor expression. **(A-C)** Widefield fluorescence microscopy of live intracellular parasites expressing the indicated PIP biosensors, classified by the DG reporter distribution phenotype: DG-less, normal (arrows) or LAP body (arrow heads). **(A)** Control reporter strains RH-GRA3 and RH-GRA2 exhibit either DG-less or normal cytoplasmic puncta profiles. **(B)** Parasites transiently co-expressing GRA3-RFP or GRA2-RFP in the face of high-affinity SidM-P4M- or FAPP1PH-based PtdIns4P biosensor co-expression (PI4P HiA) form a LAP body (arrow heads). **(C-D)** Quantification of PVs exhibiting the **(C)** GRA3-RFP or **(D)** GRA2-RFP phenotypes (DG-less, normal, LAP body) in the face of co-expression with the indicated biosensor (n = 50 PVs). Data show mean ± SEM of three independent experiments. Statistical analyses compared the control reporter strain (RH-GRA3-RFP or RH-GRA2-RFP) and parasites co-expressing the DG reporter and the appropriate PIP biosensor with regard to LAP body phenotype. Statistical significance was calculated using two-way ANOVA followed by Dunnet’s multiple comparison test; p > 0.05 (ns); p ≤ 0.05 (*); p ≤ 0.01 (**); p ≤ 0.001 (***); p ≤ 0.0001 (****). **(E)** Expression of the SidM-P4M^K568A^ and FAPP1PH^K7A/R18L^ biosensor PtdIns4P-binding mutants (PI4P HiA mut) does not affect GRA3-RFP or GRA2-RFP distribution. **(F)** Parasites co-expressing the GRA3-RFP or GRA2-RFP reporter with the lower affinity (LoA) PtdIns4P YFP-GOLPH3 biosensor do not form LAP bodies.

The control condition was represented by RH-GRA3 or RH-GRA2 strains that presented typical DG cargo distribution phenotypes (Fig 3A). It is only in rare cases (~ 1% of PVs imaged) did we detect parasites showing a LAP body-like DG distribution. In cells expressing the high affinity FAPP1PH and SidM-P4M PtdIns4P-binding domains, the DG cargo reporters were housed in LAP bodies (Fig 3B, large). Quantification of those images reported a significant exaggeration of this phenotype for both the GRA3 (mean % of PVs with LAP bodies ± SEM = 36.4% ± 3.2 for FAPP1PH and 58.5% ± 14.8 for SidM-P4M; Fig 3C) and the GRA2 reporters (mean % of PVs with LAP bodies ± SEM = 48% ± 5.2 for FAPP1PH and 64% ± 3 for SidM-P4M; Fig 3D). Thus, LAP body formation was accompanied by altered intracellular distribution of at least two DG cargos.

### DG cargo accumulation in LAP bodies reflects an intrinsic defect in PtdIns4P signaling

Induction of LAP body formation requires expression of SidM-P4M and FAPP1PH biosensors with PtdIns4P binding activity. High level expression of the mutant SidM-P4M^K568A^ and FAPP1PH^K7A/R18L^ biosensors defective for PtdIns4P-binding failed to induce LAP body formation or to perturb DG cargo profiles (Fig 3C-3E). Moreover, expression of the low affinity YFP-GOLPH3 biosensor failed to alter the intracellular distribution of DG cargo (Fig 3F).

Does the LAP body phenotype report an indirect effect of PtdIns4P limitation given PtdIns4P is a metabolic precursor of the higher order 4-OH PIPs PtdIns(4,5)P_2_ and PtdIns(3,4)P_2_? Two lines of evidence run counter to this possibility. First, the effects of high-affinity PtdIns4P-binding domain expression on parasite PtdIns(4,5)P_2_ and PtdIns(3,4)P_2_ pools were assessed. The status of those higher PIP pools was probed by 2xPHPLCδ-EGFP or YFP-TAPP1PH biosensors as a function of LAP body formation, respectively. If sequestration of PtdIns4P in LAP bodies decreases production of these PIP_2_ species, the predicted result is release of the corresponding PIP_2_ biosensors from the plasma membrane. This prediction was not supported by the data. FAPP1PH-RFP expression failed to compromise plasma membrane association of either PIP_2_ biosensor (S2A Fig). Second, the intracellular distributions of both GRA2-RFP and GRA3-RFP were visualized in parasites expressing the 2xPHPLCδ-EGFP or YFP-TAPP1PH. In neither case did PIP_2_ biosensor expression alter DG cargo distribution relative to unperturbed wild-type controls (S2B-S2D Fig).

### PtdIns4P stress compromises DG cargo exocytosis

The accumulation of DG cargo in LAP bodies suggested that expression of high-affinity PtdIns4P-binding domains compromised DG cargo exocytosis. As test, RH-WT and RH-SidM-P4M parasites were immunostained for GRA3 and the intensities of exocytosed GRA3 signal in the PV membrane (PVM) were ratioed to total GRA3 intensity in the PV as a measure of DG exocytosis efficiency. For RH-SidM-P4M cells, only parasites with GRA3-containing LAP bodies were analyzed. Acquisition parameters were normalized across all samples to minimize technical bias.

The data show a ca. 49% reduction in normalized GRA3 signal at the PVM of SidM-P4M-dependent LAP body-containing parasites relative to RH-WT (Fig 4A,4B). That the observed DG exocytic defects were the consequences of reduced PtdIns4P signaling was again indicated by the fact that the PVM GRA3/total GRA3 ratios measured for parasites expressing the PtdIns4P-binding mutant of SidM-P4M (RH-SidM-P4M^K568A^ parasites) were not significantly diminished relative to those of RH-WT (Fig 4B). These collective data demonstrate that PtdIns4P stress in *T. gondii* results in: (i) altered DG biogenesis/trafficking, and (ii) compromise of the normally efficient exocytosis of DG cargo.

**Fig 4:**
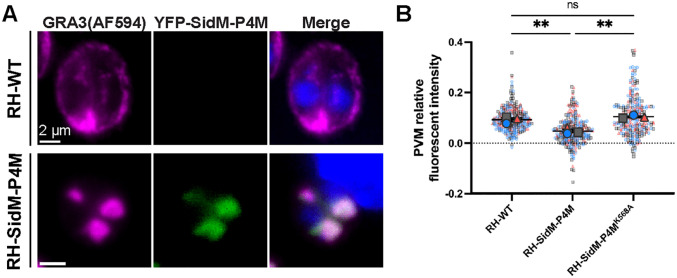
Sequestration of PtdIns4P inhibits secretion of GRA3 to the PVM. **(A)** Widefield fluorescence microscopy of fixed intracellular parasites immunostained with anti-GRA3 immunoglobulin in the absence (upper panel) or presence (lower panel) of SidM-P4M-YFP expression. In RH-WT cells, GRA3 localizes to the PV and PVM. In RH-SidM-P4M cells, GRA3 is mainly retained in the LAP body in the intracellular apical region and is absent from the PVM. Parasite DNA is visualized using Hoechst (blue). **(B)** Quantification of fluorescence intensity of GRA3 in the PVMs of parasites exhibiting SidM-P4M-dependent LAP body formation is significantly reduced compared to that of PVMs of RH-WT and SidM-P4M^K568A^– expressing strains. The box and whisker plot shows individual measurements of each PVM (n = 94; smaller symbols) per biological replicate (n = 3; orange triangles: first replicate; grey squares: second replicate; and blue circles: third replicate). The average of each biological replicate (larger symbols) was used to calculate overall average ± SEM and statistical significance. Statistical significance was calculated using one-way ANOVA followed by Tukey’s multiple comparison test; p > 0.05 (ns); p ≤ 0.05 (*); p ≤ 0.01 (**); p ≤ 0.001 (***); p ≤ 0.0001 (****).

### Ultrastructure of DG compartments under conditions of PtdIns4P stress

Two independent approaches were employed to arrive at a higher resolution description of LAP body structure as such morphological data might reveal the nature of the exocytic block. First, LAP bodies were imaged using confocal microscopy coupled with an Airyscan detector that increased spatial resolution some 1.7X and an increase in signal to noise ration of up to 8X after linear deconvolution [48]. This enhanced resolution is accurate down to 140 nm in the *xy*-plane – thereby allowing quantification of alterations in DG morphologies. In G1/S RH-WT parasites, GRA3-positive DGs were distributed throughout the cell (z-projection in Fig 5Ai; S3 Fig; S1, S2 Videos). These DGs at specific sections of a set of z-stacks exhibited a mean diameter of 255.7 ± 28.9 nm (Fig 5Aii). By contrast, the LAP bodies of RH-YFP-SidM-P4M-expressing parasites were structurally heterogeneous and were visualized as a ‘clustered’ network of smaller DGs (Fig 5Bi). These smaller DGs (sDG) exhibited a mean diameter of 153.3 ± 8.3 nm that was approximately half of that exhibited by DGs of wild-type parasites (Fig 5Bii, 5C; S3, S4 Videos). PtdIns4P-positive structures of dimensions like those of sDGs but devoid of GRA3 were also observed (mean diameter of 148.4 ± 2.9 nm; Fig 5Biii, 5C). Merge of the GRA3 and RH-YFP-SidM-P4M profiles confirmed colocalization of these two reporters (Pearson’s correlation coefficient = 0.843 ± 0.04; black arrows in Fig 5D; S3, S4 Videos). A striking feature of the z-projection images was that the PtdIns4P biosensor was not isotropically distributed throughout the LAP body. Rather, the biosensor (and by inference PtdIns4P) was concentrated in discrete domains throughout the sDG network (white arrows in Fig 5D) – potentially reflecting different classes of cargo vesicles within the LAP body. This was a reproducible result as evidenced by a biologically independent reconstruction example (S4 Fig).

**Fig 5:**
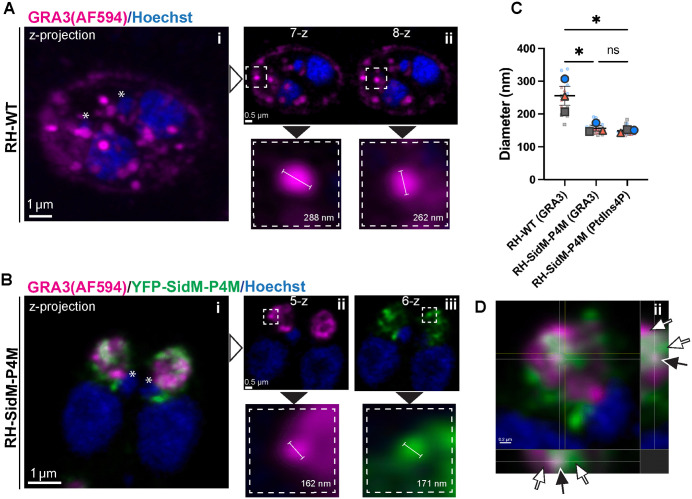
The LAP body is comprised of a network of small GRA3- and PtdIns4P-containing vesicles. **(A-B)** Z-projections of RH-WT and RH-SidM-P4M parasites stained with anti-GRA3 immunoglobulin (see S3A and S3B Fig for complete set of z-stacks, respectively). G1/S phase was identified by a single undivided apicoplast in the cells of interest (asterisk). Parasite DNA is visualized using Hoechst (blue). **(Ai)** Control RH-WT parasites showing DG cargo (GRA3) distributed in discrete puncta throughout the cytoplasm and PVM and the absence of a LAP body in the subapical area. **(Aii)** Discrete DGs were identified in Airyscan sections (example highlighted in dashed box). Diameters of 5 DGs were measured from each of three independent parasites. **(Bi)** The LAP bodies in RH-SidM-P4M parasites in G1/S phase consist of numerous GRA3-containing sDGs decorated with PtdIns4P. **(Bii)** Discrete sDGs were identified in Airyscan sections (example highlighted in dashed box). Diameters of 7-9 sDGs were measured from each of three independent parasites. **(Biii)** Discrete PtdIns4P-positive vesicles were identified in Airyscan sections (example highlighted in dashed box). Diameters of 6-9 PtdIns4P-positive vesicles were measured from each of three independent parasites. **(C)** Quantification of the diameter (nm) of DGs in RH-WT parasites (n = 5), sDGs of RH-SidM-P4M parasites (n = 7-9), and PtdIns4P-positive vesicles of RH-SidM-P4M parasites (n = 6-9). Mean values ± SEM are shown. Statistical analyses used one-way ANOVA followed by Tukey’s multiple comparison test to assess significance; p > 0.05 (ns); p ≤ 0.05 (*); p ≤ 0.01 (**); p ≤ 0.001 (***); p ≤ 0.0001 (****). **(D)** A Z-projection of the LAP body (**Di**) is shown. View sections through that Z-projection are shown in (**Dii**). Spatial co-localization of GRA3 and PtdIns4P reporters appear as white signal (indicated by black arrows). Segregation of the PtdIns4P biosensor (green) and GRA3 cargo (magenta) in distinct domains within the LAP body is clearly apparent (indicated by white arrows).

### Correlative light electron microscopy imaging

To probe LAP body morphology with higher resolution, we employed correlative light electron microscopy (CLEM). Human foreskin fibroblasts were seeded onto gridded glass-bottom coverslip dishes, infected with RH-SidM-P4M parasites, and fixed parasites were stained with antibodies directed against GRA3. The positions of either RH-WT or RH-SidM-P4M parasites were registered in the coordinate system of the gridded coverslip by confocal microscopy. RH-SidM-P4M parasites in which LAP body formation was evident, as identified by confocal microscopy, were selected as regions of interest (ROI) for CLEM imaging. The grids were then processed for transmission EM (TEM) and the ROIs visualized by TEM.

In agreement with the Airyscan imaging experiments, TEM acquisitions reported well dispersed mature DGs in WT parasites (Fig 6A). By contrast, RH-SidM-P4M-expressing parasites presented the LAP body as a single large cluster of electron dense vesicles in the apical region of the cell (green arrows in Fig 6B). Notably, the rhoptries of RH-SidM-P4M-expressing parasites were morphologically unperturbed – again emphasizing that LAP bodies and rhoptries are distinct compartments. The electron dense vesicles in RH-SidM-P4M were approximately 5X more numerous than the DGs of wild-type parasites (mean sDG count per EM section ± SEM = 3 ± 0.5 for RH-WT and 14 ± 3.7 for SidM-P4M; Fig 6C), and were about half the mean diameter of those observed in RH-WT parasites (242 ± 8.7 nm for RH-WT and 145 ± 4.7 nm for SidM-P4M; Fig 6D). Morphometric values for DGs obtained in RH-WT cells are in excellent agreement with the previous measurements of Dubey et al. [49]. CLEM analyses of RH-FAPP1PH parasites showed the ‘clustered’ sDGs of the LAP body exhibited noticeably reduced electron densities (Fig 6E). Thus, data from two independent high resolution imaging approaches converge on the conclusion that the LAP body formed upon parasite expression of high affinity PtdIns4P binding domains represented a tubulo-vesicular network of small vesicles that carry DG cargo.

**Fig 6:**
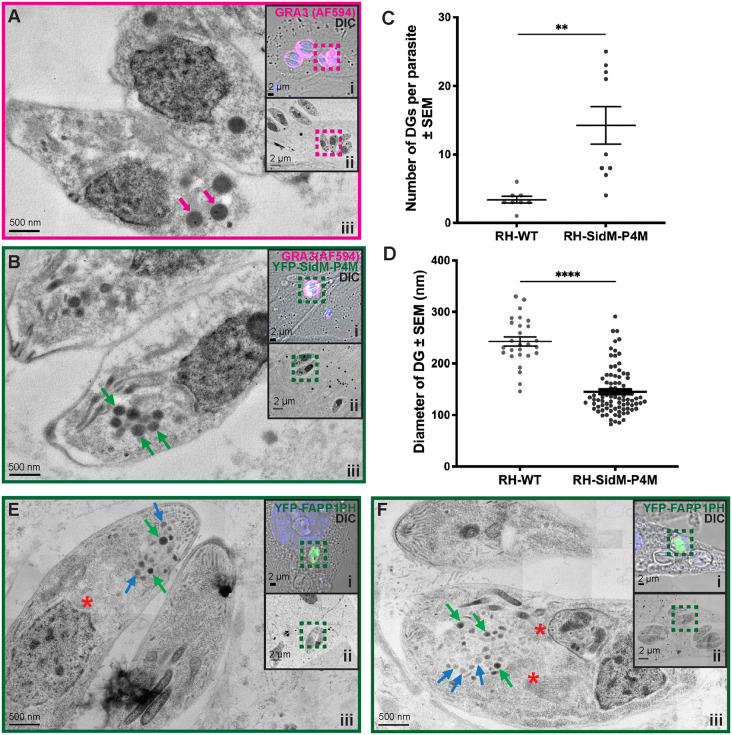
High resolution imaging of LAP body structure by correlative light electron microscopy. **(A, B and E).** CLEM acquisitions of stained RH-WT or transgenic parasites involved the following steps: (i) targeting and recognition of ROIs (dashed square) was performed in a confocal microscope and selected for imaging by TEM at (ii) low and (iii) high magnification. **(A)** RH-WT show scattered distribution of mature highly electro-dense DGs distributed throughout the cytoplasm (magenta arrows). **(B)** RH-SidM-P4M show a pool of DG-cargo carrying vesicles in the subapical area that are of smaller size (sDGs) relative to WT DGs (green arrows). **(C)** Quantification of DG number per tachyzoite and per EM section (n = 8 for WT and SidM-P4M). Data represent the mean ± SEM. **(D)** Quantification of the average DG diameter (nm) per tachyzoite and per EM section (n = 8 for WT and SidM-P4M and n = 3 for FAPP1PH). Data are represented as mean ± SEM, and statistical significance was determined using the Mann-Whitney t-test; p > 0.05 (ns); p ≤ 0.05 (*); p ≤ 0.01 (**); p ≤ 0.001 (***); p ≤ 0.0001 (****). **(E)** An RH-FAPP1PH parasite undergoing endodyogeny (red asterisks indicate position of daughter cells) presents a LAP body consisting of an ‘clustered’ sDG network (green arrows) that resides in the subapical region of the mother cell. sDGs with reduced electron density are indicated by blue arrows.

### A regulatable system for LAP body formation

To address questions related to LAP body physiology, the TIR1-mediated degron system [50] was adapted for controlled degradation of SidM-P4M. Clones expressing SidM-P4M-YFP-mAID were selected from a TIR1-expressing parasite population. In those cells, the SidM-P4M chimera is targeted for rapid proteolytic degradation upon indole acetic acid (IAA) addition to the medium and the selection was executed in IAA-replete media (IAA+) to minimize any unintended selection bias due to constitutive expression of SidM-P4M. Following antibiotic selection and cloning by limiting serial dilution, two clones (C4 and G10) were chosen for detailed study. Both elaborated SidM-P4M-positive puncta consistent with LAP bodies at 24-hours after removal of IAA from growth medium and the large SidM-P4M-positive puncta contained DG cargo (GRA3; Fig 7A). Some 60% of SidM-P4M-positive cells presented LAP bodies in this condition (Fig 7B) —as was also the case in the transient transfection experiments. In parasites cultured in the presence of IAA, however, the LAP body was rarely detected (~ 2% of counted PVs). A similarly low frequency was observed in RH-WT parasites.

**Fig 7:**
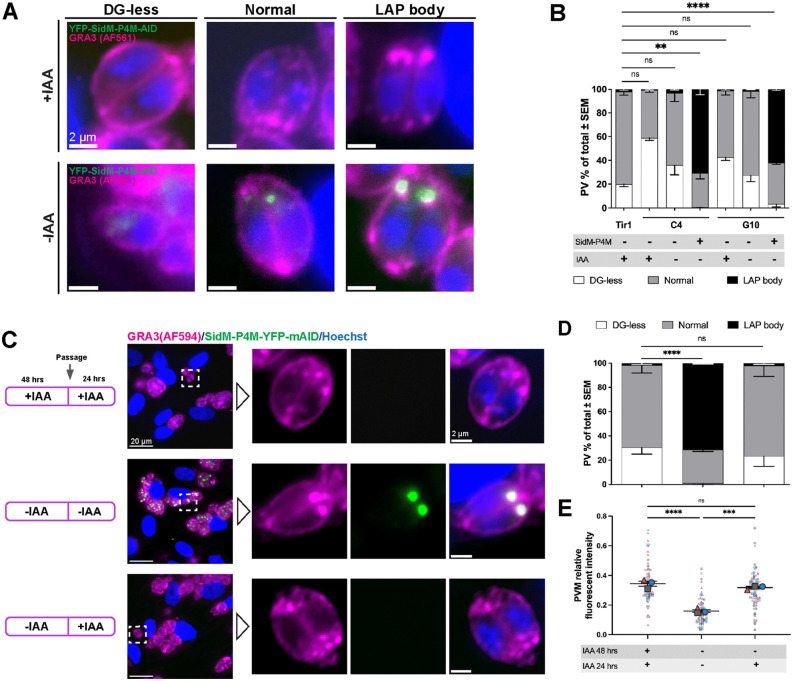
Formation and consumption of the LAP body using an IAA-activated degron system. **(A)** Widefield fluorescence microscopy of non-induced (IAA-treated) and induced (IAA-non-treated) expression of SidM-P4M-YFP-mAID in clone G10 parasites after 24 hrs. Parasite phenotypes are classified by the DG reporter distribution profile: DG-less, normal (arrows) or LAP body. Parasites were fixed and stained with GRA3 (AF568). **(B)** Quantification of GRA3 phenotypes (DG-less, normal or LAP body) observed at 24 hrs post-treatment (+IAA or -IAA) in clones G10 and C4 with 100 PVs analyzed for each condition. The SidM-P4M bar indicates detectable (+) or undetectable (-) expression of the biosensor (Sidm-P4M-YFP-AID) in PVs. **(C)** The LAP body is not a dead-end compartment. Widefield fluorescence microscopy of intracellular RH-SidM-P4M-YFP-mAID parasites (clone G10) pre-treated for 48 hrs —with or without IAA— and post-treated for 24 hrs -- with or without IAA. A pre-formed LAP body is consumed and its content secreted upon removal of the Sidm-P4M-YFP-AID PtdIns4P clamp. This chase indicates the LAP body as a functional intermediate compartment in the DG secretory pathway. **(D)** Quantification of PVs binned according to GRA3 phenotype (DG-less, normal or LAP body) in clone G10 following chase (n = 100 PVs each clone). **(B and D)** Data show the mean ± SEM of three independent experiments. Statistical analyses compared the LAP body phenotypes of the control reporter strains (RH-GRA3-RFP or RH-GRA2-RFP) and parasites co-expressing the DG reporter and the appropriate PIP biosensor. Statistical significance was calculated using two-way ANOVA followed by Dunnet’s multiple comparison test; p > 0.05 (ns); p ≤ 0.05 (*); p ≤ 0.01 (**); p ≤ 0.001 (***); p ≤ 0.0001 (****). **(E)** Quantification of fluorescence intensity of GRA3 in the PVMs of clone C4 following chase. Images used for quantification were obtained by confocal microscopy. The box and whisker plot shows individual measurements of each PVM (n = 24-33; smaller symbols) per biological replicate (n = 3; orange triangles: first replicate; blue squares: second replicate; and grey circles: third replicate). The average of each biological replicate (larger symbols) was used to calculate overall average ± SEM and statistical significance. Statistical significance was calculated using one-way ANOVA followed by Tukey’s multiple comparison test; p > 0.05 (ns); p ≤ 0.05 (*); p ≤ 0.01 (**); p ≤ 0.001 (***); p ≤ 0.0001 (****).

To develop a time-course for LAP-body formation, clone C4 and G10 parasites were allowed to infect host cells for 24hrs under +IAA conditions after which IAA was removed (t = 0) and parasites imaged at selected time points thereafter. Little SidM-P4M-YFP-mAID fluorescence signal was detected at 2hrs following IAA washout, and LAP bodies were first observed at 5hrs post-washout.

### Parasites tolerate LAP body formation

Does LAP body formation and accumulation of DG cargo within this structure compromise parasite viability? The regulatable system enabled a direct interrogation of this question. Clone C4 and G10 SidM-P4M-YFP-mAID parasites were cultured under IAA + or IAA- conditions for 4 passages (one week), with cells from the final passage fixed and imaged 24 hours post infection. Although SidM-P4M expression was evident in the IAA- condition for the duration of the experiments, the parasites appeared to grow and divide normally (S5A Fig). Moreover, these egressed at the same time points as their IAA+ control counterparts. This was the case even though nearly all parasites expressed SidM-P4M and ~90% of those presented LAP bodies (S5B Fig). These results were observed for both C4 and G10 SidM-P4M-YFP-mAID parasite clones.

### The LAP body is operationally a functional intermediate in DG biogenesis

What is the fate of DG cargo that accumulates in the LAP body? Given DG cargo is not efficiently secreted from the LAP body, one possibility is that this compartment is a trafficking ‘dead-end’. The second possibility is that the secretory defect reports a kinetic defect in DG biogenesis/trafficking and that the LAP body is a compromised intermediate in this process whose formation reflects a ‘traffic jam’ at that stage. These two possibilities can be distinguished in a chase experiment that monitors the fate of DG cargo after release of the LAP body from PtdIns4P sequestration by SidM-P4M. The former possibility predicts the accumulated DG cargo will not be secreted during chase whereas the latter predicts it will.

As test, clone C4 and G10 SidM-P4M-YFP-mAID parasites were cultured for one full infection cycle (48 hours) in the absence of IAA to allow SidM-P4M expression and LAP body formation. These parasites were then passaged to new hosts with IAA in the medium as variable. The IAA+ condition represented the chase condition where SidM-P4M-YFP-mAID is rapidly degraded and the IAA- condition served as control where the cellular load of SidM-P4M-YFP-mAID was preserved. After 24 hours of IAA addition we found SidM-P4M-YFP-mAID was no longer detectable in the parasites as determined by loss of YFP fluorescence, and that the GRA3 dense granule cargo was cleared from its anterior site of accumulation (Fig 7C, 7D). The GRA3 secretion efficiency measured in this chase condition confirmed that the cleared DG cargo remains competent for efficient exocytic secretion (Fig 7E). These data indicate the LAP body is not an exocytic dead-end but operationally a trafficking intermediate as the structure can be captured and subsequently consumed during chase.

### PtdIns4P biosensor distribution through the parasite cell cycle

PtdIns4P distribution profiles were followed throughout the parasite cell division cycle. For those experiments, an inner membrane complex (IMC) reporter was constructed where the IMC marker IMC1 was fused to RFP (IMC1-RFP) to landmark cell cycle stages in proliferating intracellular parasites – as described previously [51]. Live parasites expressing IMC1-RFP and YFP-SidM-P4M were imaged by widefield fluorescence microscopy during various stages (S6 Fig). Representative images collected for each cell cycle stage are shown in Fig 8A. In G1/S parasites, YFP-SidM-P4M localized primarily to the LAP body in agreement with the various data documented above. At initiation of cell division, a prominent recruitment of YFP-SidM-P4M to a split juxta-nuclear stacked compartment was observed (white arrows in Fig 8Ai) and, to a lesser extent, to apical vesicles (yellow arrows in Fig 8Ai). Quantification of parasites where YFP-SidM-P4M was concentrated on the LAP body demonstrated that, contrary to what was observed in G1/S parasites or for cells in the next division stage (elongation 1), only ~8.5% of cells at the initiation phase exhibited positive signal at the LAP body. The remaining cells exhibited profiles that reported redistribution of YFP-SidM-P4M to the split juxta-nuclear stacked compartment (Fig 8Aii, 8B). During cell elongation stages, that stacked compartment completed fission and each daughter cell inherited a copy. Thus, the split juxta-nuclear compartment exhibited the features expected of a dividing Golgi system [52]. That this stacked compartment indeed represented the Golgi/TGN was confirmed by the fact that it was marked with TgSORTLR (Fig 8C).

**Fig 8:**
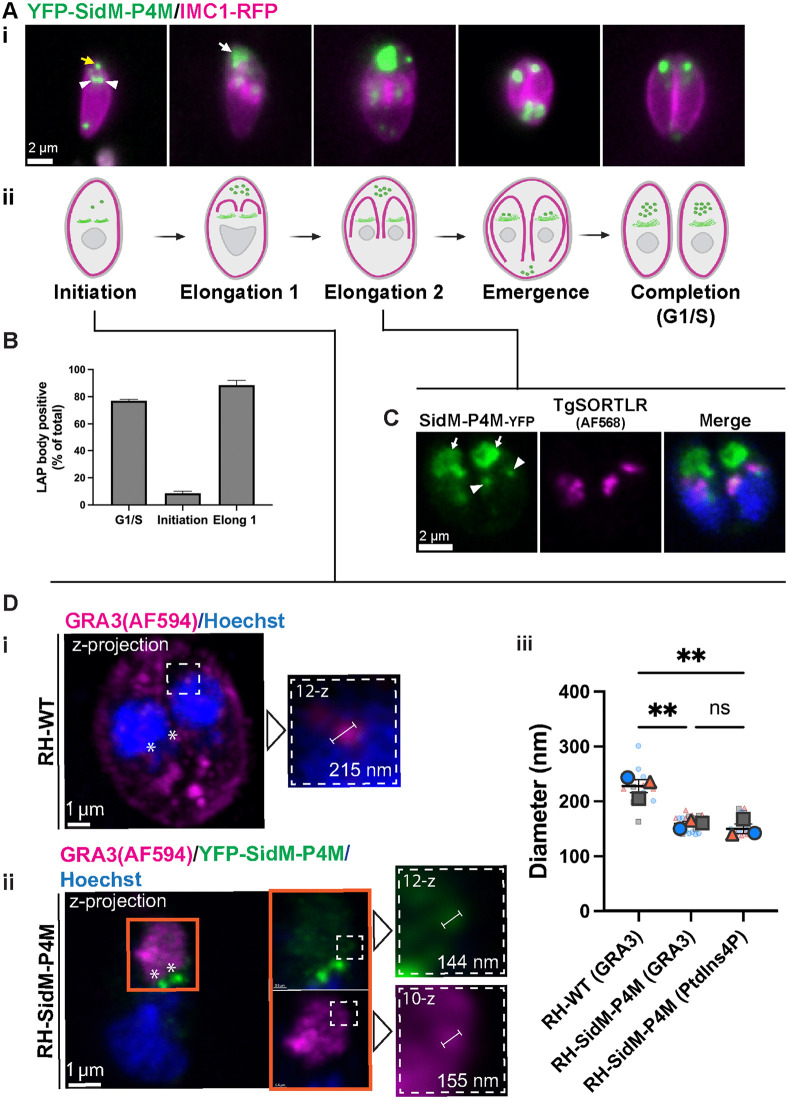
PtdIns4P biosensor relocates away from the LAP body during the initial stages of parasite cell division. **(Ai)** Widefield fluorescence microscopy images of live cells co-expressing YFP-SidM-P4M and cell cycle marker IMC1-RFP at each step of the cell cycle are shown. A dividing Golgi system marks initiation of endodyogeny (arrowheads). YFP-SidM-P4M targets to the LAP body during most stages of the cell cycle (white arrows). During the initiation stage, however, YFP-SidM-P4M redistributes to the dividing Golgi system (arrowheads) and to apical vesicles (yellow arrow). **(Aii)** Schematic representation of the relative positions of the IMC (magenta) and of PtdIns4P pools (green) at the Golgi/TGN and post-TGN compartment at each cell cycle stage (initiation, elongation (1 and 2), emergence and completion/G1). Created in BioRender. https://BioRender.com/06jf4im **(B)** Quantification of parasites showing recruitment of YFP-SidM-P4M to the LAP body at the indicated stages of endodyogeny. Quantification data were obtained from two independent experiments (n ≤ 15 parasites per stage). Data show mean ± SEM. **(C)** Confocal IFA images identifying Golgi/TGN (TgSORTLR) in a dividing RH-SidM-P4M parasite during the elongation stage of cell division. YFP-SidM-P4M targets to the dividing Golgi/TGN compartment (arrowheads) and the LAP body (arrows). DNA is visualized using DAPI (blue). **(D)** Z-projection of parasites immunostained with anti-GRA3 immunoglobulin as these initiate endodyogeny (see **S4****A,** **S4B Fig** for complete z-stack sets). This stage is identified by the division of apicoplast in RH-WT (DNA visualized with Hoechst; asterisks) and fission of the Golgi/TGN system in RH-YFP-SidM-P4M cells (asterisks). Discrete DGs were identified in Airyscan sections (example highlighted in dashed box). Diameters of 4-9 DGs were measured from each of three independent parasites. Parasite DNA is visualized using Hoechst (blue). **(Di)** Control RH-WT parasites show GRA3 distributed in discrete puncta throughout the cytoplasm and PVM. **(Dii)** The RH-YFP-SidM-P4M strain displays a GRA3 positive-LAP body with diminished YFP-SidM-P4M signal (proxy for PtdIns4P). YFP-SidM-P4M preferentially targets to the recently divided Golgi/TGN system (orange square). **(Diii)** Quantification of the diameter (nm) of DGs in dividing RH-WT parasites (n = 4-6), sDGs of dividing RH-SidM-P4M parasites (n = 7-9), and PtdIns4P-positive vesicles of dividing RH-SidM-P4M parasites (n = 7-8). Data are presented as mean ± SEM, and statistical significance was determined using one-way ANOVA followed by Tukey’s multiple comparison test; p > 0.05 (ns); p ≤ 0.05 (*); p ≤ 0.01 (**); p ≤ 0.001 (***); p ≤ 0.0001 (****).

By contrast to the Golgi system, the LAP body was neither divided nor inherited. At elongation stages of the cell cycle, YFP-SidM-P4M partially re-localized back to the LAP body of the mother cell and, upon daughter cell emergence, the LAP body migrated towards the basal pole of mother cell body remnants -- potentially to the residual body. At completion of the cell cycle (marked by entrance of daughter cells into G1), YFP-SidM-P4M redistributed primarily to a LAP body profile. Thus, the biosensor data report a mobilization of the PtdIns4P biosensor from the LAP body to a newly dividing Golgi system upon initiation of endodyogeny, and relocalization of the biosensor back to the LAP body in subsequent stages of the cell cycle. We noted that all three PtdIns4P biosensors also marked small cytoplasmic puncta that frequently had a localization consistent with the residual body in tachyzoites that had completed the first round of cell division post-infection (Fig 8i).

### PtdIns4P biosensor redistributes from the LAP body in dividing parasites

The ‘disappearance’ of the PtdIns4P-positive LAP body in parasites initiating cell division, suggested two possibilities: (i) the LAP body is disassembled during initiation of endodyogeny and reforms in later stages of the cell cycle, or (ii) the biosensor relocates away from an otherwise intact LAP body. To distinguish between these possibilities, LAP body structure was examined in dividing parasites by high resolution Airyscan confocal imaging, with the modification that GRA3 replaced YFP-SidM-P4M as LAP body marker. In RH-WT parasites, GRA3-positive DGs were distributed throughout the cell during the initiation of mitosis as landmarked by the elongation of the apicoplast (asterisk in Fig 8Di; [53]). By contrast, parasites expressing YFP-SidM-P4M during the initiation of cell division exhibited a GRA3-positive LAP body (Fig 8Dii; S5, S6 Videos). Whereas RH-WT DGs exhibited a mean diameter of 227 ± 11.7 nm – a value consistent with that measured for DGs of RH-WT cells in G1/S phase (255 ± 28.9 nm; see above) -- the sDGs that comprised the LAP body in those dividing parasites presented dimensions similar to those of YFP-SidM-P4M-expressing parasites in G1/S phase (mean ± SEM in G1/S phase: 156 ± 8.3 nm vs in mitosis: 158 ± 4.5 nm) (Fig 8Dii, 8Diii). This GRA3-positive structure remained prominent even though SidM-P4M PtdIns4P biosensor localization to the LAP body was strongly diminished during initiation of cell division (Pearson’s correlation coefficient 0.486 ± 0.07 vs 0.843 ± 0.04 for G1/S phase cells; Fig 8Dii). Thus, YFP-SidM-P4M redistributes from the LAP body to the Golgi/TGN system at the initiation of cell division and returns to the LAP body in subsequent stages of the cell cycle.

## Discussion

Current models envision formation of DGs to result via direct budding from the *T. gondii* TGN as mature structures with appropriately sorted and condensed cargo [30]. Thus, DGs are not considered to undergo the types of more complex maturation processes that are hallmarks of dense core vesicle compartments that form the basis of regulated exocytosis systems of other eukaryotes – e.g., dense core granules of neuroendocrine cells or insulin secretory granules (ISGs) of pancreatic β-cells. Direct budding models are based on imaging studies that document the presence of morphologically uniform mature DGs with no recognizable maturation intermediates [54,55].

Direct budding mechanisms leave unanswered questions, however. Although there is considerable evidence to suggest that the DG exocytic pathway is a default pathway in *T. gondii* [56,57], these models nevertheless require that DG cargo be segregated from other secretory cargo and condensed —presumably at the site of DG budding from the TGN surface. Moreover, proteins marked for retention in the TGN must either be excluded from loading into the nascent DG vesicle or otherwise retrieved from a DG vesicle after scission from TGN membranes. How appropriate cargo is condensed into these electron dense granules remains an unresolved question in the field of apicomplexan secretory trafficking. Mechanisms of cargo quality control are also not understood in detail. These problems are of particular interest as efficient trafficking and exocytosis of virulence factors is fundamental to host cell invasion by the parasite, its intracellular proliferation, and ultimate egress of the parasite from the exhausted host cell to initiate successive rounds of infection.

### Reconsidering ideas for DG biogenesis

Our findings indicate that DG formation in *T. gondii* is a more complex process than that envisioned by simple direct budding models – one more in line with the pathways of dense core vesicle and secretory granule biogenesis in other systems. Using neuroendocrine cells as example, dense core granules mature in a stage-specific progression of homotypic fusion and retrograde trafficking of missorted cargo back to the TGN [58]. Moreover, the maturation process is typically slow in systems where immature DG-like structures are visible under the electron microscope. For example, insulin secretory granule (ISG) biogenesis in mammalian pancreatic β-cells is a high flux pathway, but ISG maturation requires three hours to complete [59,60]. The lack of information regarding discrete stages of DG biogenesis in *T. gondii* might reflect the process being too rapid and/or of insufficient flux to permit visualization of transient biogenic intermediates by electron microscopy —i.e., the only method with sufficient resolution to confidently recognize such intermediates.

As demonstrated in other systems [61,62], in vitro reconstitution or in vivo stage-specific inhibition approaches are required to systematically dissect DG biogenesis and arrive at a detailed description of the process in *T. gondii*. We posit that expression of high affinity PtdIns4P binding domains in *T. gondii* is informative from the latter perspective. LAP body morphology as a ‘clustered’ network of small PtdIns4P-decorated vesicles loaded with DG cargo argues for a more complex process for DG biogenesis in *T. gondii*. Furthermore, induction of LAP body formation by high affinity PtdIns4P biosensor expression demonstrates a previously unappreciated role for PtdIns4P signaling in DG biogenesis. Finally, the morphological properties of the LAP body offer insights into this process. In Fig 9 we outline a model for DG biogenesis that takes these insights into account.

**Fig 9:**
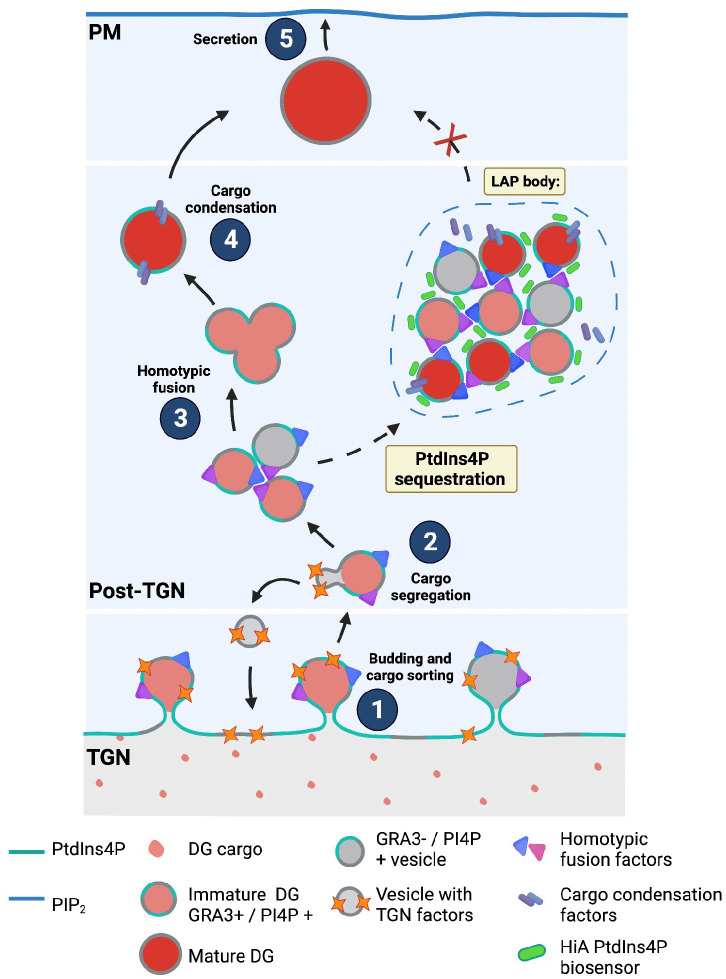
Model for DG biogenesis, maturation and exocytosis. Solid arrows mark proposed model for DG biogenesis and maturation in *T. gondii*. Budding and cargo sorting from PtdIns4P-enriched domains of the TGN (**step 1**) are followed by a further round(s) of cargo segregation (**step 2**) to generate immature DGs. Maturation of these structures at a post-TGN compartment is characterized by the PtdIns4P-dependent homotypic fusion (**step 3**) —interference of which results in LAP body formation. Subsequent steps involve cargo condensation in the maturing DGs (**step 4**). Removal of PtdIns4P from mature DGs membrane marks completion of the maturation process and acquisition of secretion competence (**step 5**). Created in BioRender. https://BioRender.com/06jf4im.

### Functional interpretation of the LAP body

What does the LAP body represent and how does this structure relate to DG biogenesis? We favor the idea that the LAP body represents an exaggerated compartment of nascent DGs retarded at a late stage(s) of biogenesis -- one we loosely refer to as maturation. The morphological data indicate that the small DG cargo-containing vesicles that comprise the LAP body network have completed budding from the TGN (Fig 9, step 1). Those data further suggest the process of cargo segregation has largely been completed (Fig 9, step 2). These conclusions are supported by demonstrations that the small LAP body vesicles are loaded with multiple DG cargo, are of homogeneous dimensions and are devoid of detectable quantities of TGN resident proteins (e.g., TgSORTLR). Thus, the small vesicles are not a result of TGN fragmentation induced by expression of high affinity PtdIns4P binding modules. That these vesicles are smaller and frequently less electron dense than the mature DGs of WT parasites suggests a late maturation step(s) is perturbed. In turn, these perturbations compromise secretion of DG cargo. The LAP body trafficking defect is not a terminal one. Rather, it exhibits the hallmarks of a kinetic block given DG cargo sequestered in LAP bodies chase upon release of the PtdIns4P clamp set by high affinity binding protein expression.

### A role for PtdIns4P in DG biogenesis/maturation

In sum, we hypothesize the LAP body represents an exaggerated form of a bona fide late secretory intermediate. Such interpretation of the data demands that the ‘wild-type’ version of this intermediate be a very transient structure —one rapidly formed and rapidly resolved into mature DGs. That we observe LAP body-like structures in ~1–2% of unperturbed parasites is consistent with this general idea and prompts further study. We interpret the exaggerated LAP body to be a manifestation of insufficient PtdIns4P signaling in the process of DG biogenesis/maturation —a deficit induced upon PtdIns4P sequestration by the high-affinity binding modules. The small dimensions of the clustered LAP body vesicles relative to mature DGs of WT cells suggest that homotypic fusion of the small clustered DGs is a discrete PtdIns4P-requiring step in the formation of the larger mature DGs (Fig 9, step 3). That PtdIns4P distribution within the LAP body and on the small vesicle surfaces is not isotropic reports the existence of discrete PtdIns4P domains on clustered DGs. We speculate these domains concentrate vesicle factors required for homotypic tethering and/or fusion as in the vacuolar homotypic fusion system discovered by [63]. In that regard, mammalian COPII coated vesicles derived from the endoplasmic reticulum engage in homotypic tethering to form vesicular/tubular clusters [VTCs; 64].

Although not known to be a PtdIns4P-dependent process for COPII vesicles, the general morphological similarities in the VTCs and LAP bodies are notable. Given that the LAP body DGs are frequently less electron dense than the mature DGs of WT parasites, PtdIns4P might also be required for the optimal activity of factors required for cargo condensation (Fig 9, step 4). Cargo condensation in dense core granules of other systems requires recruitment of proton pumps into the maturing DG to acidify the compartment and potentiate cargo packing into crystalline forms [65,66]. The existence of PtdIns4P-positive structures in the LAP body devoid of GRA3 cargo suggest different classes of vesicles populate the network. Fusion of immature DGs with these vesicles might contribute to delivery of factors that regulate DG cargo condensation.

### Signals for completion of DG maturation and competence for exocytosis

Models positing a PtdIns4P signaling involvement in DG maturation must account for how completion of maturation is recognized. In that regard, LAP-body DGs are marked with both PtdIns4P and DG cargo, whereas mature DGs do not recruit high affinity PtdIns4P biosensors. We infer from those data that the cytosolic surfaces of mature DG membranes are depleted for PtdIns4P, and that this loss marks completion of the maturation process and acquisition of competence for final secretion (Fig 9, step 5). Such a strategy applies to the maturation pathway of post-Golgi secretory vesicles in yeast where the PtdIns4P loaded during the budding process is degraded as the vesicles approach sites of exocytosis – a lipid remodeling event that reprograms the small GTPase specificity of vesicle-associated nucleotide exchange factors [67]. PtdIns4P pools on insulin secretory granule surfaces must also be degraded by the PtdIns4P-phosphatase Sac2 prior to mature granule association with the mammalian plasma membrane [68,69].

Regarding regulation of PtdIns4P levels, we observed that high affinity PtdIns4P biosensors significantly redistributed away from the LAP body to the dividing Golgi system in parasites initiating endodyogeny. This redistribution from the LAP body to the Golgi/TGN system and biosensor return in subsequent stages of the cell cycle, suggests a complex and previously unappreciated reprogramming of PtdIns4P signaling in DG cargo-containing compartments upon initiation of endodyogeny. One idea is that such reprogramming reflects a shift in emphasis from cargo exocytosis to Golgi division and partitioning during endodyogeny

### PtdIns4P signaling in apicomplexans

Apicomplexa are excellent model organisms for the study of adaptations relevant to parasitic lifestyles. As such, understanding the origin and landscape of PIP signaling in Apicomplexa defines an interesting point of comparison relative to PIP signaling strategies in other eukaryotes [29]. This study identifies a role for PtdIns4P in DG maturation and ultimate exocytosis of DG cargo in *T. gondii*. The similarities to aspects of both constitutive and regulatory secretory pathways in other eukaryotes are noteworthy as described above. This is perhaps not surprising as *T. gondii*, given its parasitic lifestyle, can be considered a professional secretory cell —one for which there is evidence for both constitutive and regulated DG secretory pathways [30,55,70].

The collective data raise key questions regarding: (i) which PtdIns 4-OH kinase(s) produces the PtdIns4P pool(s) whose sequestration induces LAP body formation and defects in DG exocytosis, and (ii) how is synthesis of this PtdIns4P pool regulated? Regarding the latter, LAP body phenotypes largely recapitulate those recently described in mammalian pancreatic β-cells where various stages of insulin secretory granule formation, maturation and regulated exocytosis are PtdIns4P-dependent processes whose execution is perturbed upon functional ablation of PITPα —a PtdIns/phosphatidylcholine transfer protein (PITP) that stimulates PtdIns4P production in pancreatic β-cell TGN membranes [71]. Similarly, the major yeast PITP Sec14 plays an essential role in potentiating PtdIns4P-dependent membrane trafficking through the TGN/endosomal system [11,12,72–75].

Does *T. gondii* utilize a PITP in a similar manner for PtdIns4P-dependent DG activities? This seems probable as *T. gondii* potentially encodes ten Sec14-like PITPs and one mammalian PITPα-like ortholog (ToxoDB (v56); http://ToxoDB.org). Moreover, time-resolved phospho-proteome analyses identify a Sec14-like protein (TGME49_254390) and a putative PtdIns 4-OH kinase (TGME49_276170) in a cohort of lipid signaling proteins phosphorylated upon induction of egress —a process dependent on phosphatidic acid and phospholipase C signaling [76,77]. Yet another T. gondii Sec14-like protein (TGME49_213790) is appended to a PH domain —as is the single PITPα-like ortholog (TGME49_289570). Such domain architectures recommend these putative PITPs as attractive candidates for engaging the PtdIns4P pool(s) identified herein. Functional dissection of these activities promises to clarify the mechanism by which PtdIns4P signaling regulates DG biogenesis, maturation and exocytosis in apicomplexan parasites.

## Materials and methods

### Cell culture and *T. gondii* strains

The human foreskin fibroblast-1 (HFF-1) cell line was obtained from the American Type Culture Collection and grown in Dulbecco’s modified Eagle’s medium with 10% fetal bovine serum (FBS) and incubated at 37°C with 5% CO_2_ in a humidified atmosphere. The *T. gondii* RH strain was maintained by serial passage using HFF-1 monolayers as host cells. Parasites were cultured to egress and purified from host cells by disrupting the host cells via serial passage through 18- and 25-guage needles. Parasites were subsequently collected from the host cell lysate in the pellet after centrifugation of the crude lysate at 900 x g for 5 minutes.

### Transient transfection and selection of stable transgenic parasites

RH strain was transfected by electroporation as described previously [78]. Using an electroporation cuvette as vessel, 10^7^ tachyzoites were mixed with 25 μg of plasmid in transfection buffer (120mM KCl, 150μM CaCl_2_, 5mM MgCl_2_, 2mM EDTA, 25mM HEPES KOH, 10mM potassium phosphate, pH 7.6). In co-transfection experiments, 12.5 μg of each plasmid was used. A single electrical pulse (1.3kV, 25uF) was applied using the Bio-Rad Gene Pulse II electroporation apparatus. The mix of total transient and stable transfected parasites were allowed to recover after plating on an HFF-1 monolayer in the absence of drug for 24 hours and then immediately imaged. When appropriate, the selection of stable transgenic parasites was performed to obtain stable RH-SidM-P4M, RH-Fapp1PH, and RH^Tir1^-SidM-P4M and strains, in the presence of a selection drug: chloramphenicol (20 μM) or mycophenolic acid (25 μg/ml) with xanthine (25 μg/ml). Then, stable clones were isolated by limiting dilution under conditions of continuous drug selection. For the degron experiment in RH TIR1–3FLAG cells (BEI Resources NR 51145), 10 clones were tested for responsiveness to IAA removal, for degree of SidM-P4M expression, and for stability of the SidM-P4M transcript over 4 passages.

### Plasmid constructs

All *T. gondii* expression vectors expressing fluorescently-labeled organelle markers were based on the pTUB-CAT (Chloramphenicol acetyltransferase) system [79]. Gene expression is driven by the *tubulin* promoter. In all cases, the DNA restriction fragments carrying the gene of interest were subcloned into the BglII/AvrII sites of pTUB. These constructs were engineered such that these carried a myc-tag and a fluorescent protein gene between flanking the cloning site. Additional details are described in the Figure legends. The primers used for cloning all *T. gondii* biomarkers are listed in S1 Table. The SidM-P4M gene was amplified from the GFP-P4M-SidM plasmid gift from Tamas Balla; Addgene plasmid # 51469; RRID:Addgene_51469). For degron experiments, the pTUB1:YFP-mAID-3HA, DHFR-TS:HXGPRT was provided by David Sibley (Addgene plasmid # 87259; RRID:Addgene_87259).

### Immunofluorescence analysis (IFAs)

Confluent HFF-1 host cells grown in 35mm glass-bottom coverslip dishes were infected with tachyzoites for 24 hrs, washed 1X with phosphate buffered saline (PBS) 1X and then fixed with 4% PFA (v/v) for 15 min at room temperature and washed 3X (all washing incubations were performed for 10 min with PBS 1X solution). Cells were permeabilized with 0.2% Triton-X (v/v) for 4 min at RT, blocked with 2% BSA in PBS 1X overnight at 4°C, incubated with organelle-specific primary antibodies diluted in blocking buffer for 1 hour. Details regarding the primary and secondary antibodies used in this study are listed in S2 Table. Coverslip were washed with PBS three times, incubated with conjugated secondary antibodies listed in S2 Table in blocking buffer for 1 hour, and washed again with PBS three times. Cell DNA was stained with Hoechst solution (1:5000) for 5 min. After three washes with PBS, coverslips were flooded with PBS and stored at 4˚C for no longer than seven days.

### Image acquisition

Confocal images were collected in a NikonA1R microscope (CFI Plan Apo lambda 60x/1.4 oil objective) and Zeiss LSM 780 NLO multiphoton microscope (Plan-Apochromat 63x/1.4 oil DIC M27 objective). Imaging processing was performed using ImageJ (NIH). Pearson’s correlation coefficients were calculated using the colocalization analysis plugin JaCoP in ImageJ and with the colocalization analysis tool in Zeiss ZEN Blue 2.3.

Super-resolution fluorescence imaging was performed with a Zeiss LSM 780 confocal microscopy with 32 channel GaAsP array for detection of multicolor-labeled samples and a Airyscan super-resolution detector. A Plan-Apochromat 63x/1.4 oil DIC M27 objective and continuous wavelength laser lines at 405 (Hoechst), 488 (YFP), and 561 (AF594) nm were also used for acquisition. The optimum Z-step size between stacks (159.5 nm), x and y resolution (20.4518 pixels/μm), and image size (920x920 pixels) were automatically set by the Zeiss Zen (Black edition) software upon selection of lasers and gain (850). ROI in control samples consisted in RH-WT parasites labeled with anti-GRA3, while ROI in experimental samples were defined as RH-SidM-P4M parasites containing a LAP body stained with anti-GRA3. The acquired z-stacks were deconvoluted using Zeiss ZEN 2.3 (blue edition) software, and gamma values were increased or decreased in the order of 0.1-0.8 units. Z-projections and section views were generated with Imaris 9.8v. The measurement of stained vesicles diameter was performed after the selection of the larger, single, and circular stained vesicle without rough edges at a specific section among a set of z-stacks. Diameter measurements were calculated using the measurement tool in ImageJ. Pearson’s correlation and colocalization coefficients of regions of interest (subapical areas of individual tachyzoites) using the JACoP plugin in ImageJ.

### Definition and quantification of DG profiles

Transgenic parasites were generated by electroporation as described above. Two plasmid pTUB constructs were used for co-transfection: 12.5 μg of pTUB-Myc-GRA3/2-RFP and 12.5 μg of pTUB constructs expressing YFP-FAPP1PH, YFP-SidM-P4M, YFP-GOLPH, YFP- SidM-P4M^(K568A)^, FAPP1PH^(K7A/R18L)^, 2xPHPLCδ-EGFP, or TAPP1PH-YFP as appropriate. Transfected tachyzoites were plated onto confluent HFF-1 cells in 35mm glass-bottom coverslip dishes with 2 ml of supplemented DMEM. For transient experiments, plates were incubated for 24 hours. Only parasites expressing the constructs of interest were counted using an epifluorescence microscope (Nikon Eclipse T*i*). The criteria used for binning parasite populations based on GRA3 and GRA2 phenotype was as follows: 1) Each unit was considered as a PV that contained one or more tachyzoites; 2) up to 50 PVs were counted per experiment; 3) PVs were binned as displaying large or small puncta phenotype when at least one of the tachyzoites in the vacuole presented one of these phenotypes. In cases where both small and large puncta pattern were observed in the same PV, the PV was classified as displaying large puncta. All experimental results represent the data from three independent biological replicates. For TIR1-mediated degron experiments, selected clones expressing pTub-SidM-P4M-YFP-mAID in the RH-TIR1 background were allowed to infect confluent HFF-1 cell monolayers in the presence or absence of IAA for the indicated times. The same imaging and quantification protocol was subsequently followed.

### Ratiometric measurement of DG exocytosis efficiency

Wild-type and transiently transfected parasites were grown for 24 h, fixed with 4% PFA and stained with anti-GRA3 as mentioned previously. Imaging and counting of 100 PVs per experimental and control conditions was performed in epifluorescence mode. Camera settings such as format (no binning), exposure time (10 msec) and dynamic range (11-bit and Gain 4) were fixed for the TRITC filter channel to collect unbiased imaging data for all experimental conditions. To calculate GRA3 secretion efficiency as a function of transient YFP-SidM-P4M expression, we adopted the ratiometric approach of Li et al [80]. For the degron experiments, images were obtained by confocal microscopy. Imaging parameters were set to eliminate over- and underexposure and kept consistent for all samples within an experiment within the red channel (laser power 5.00, HV 83, offset -3, pinhole 1.2AU). ROI were drawn around the PVM region (PVM), the intracellular region occupied by the nucleus (nuclear region), the area surrounding the entire PV and parasites (Total *Toxoplasma*), and a background region outside the PV. The mean intensities for each region were recorded with the ImageJ measurement tool and the PVM relative intensity was expressed as:


$$\begin{array}{c} PVM\;relative\;intensity=\frac{(mean\;PVM-mean\;nuclear\;region)\;x\;PVM\;area}{(mean\;total\;Toxoplasma-mean\;background)\;x\;area\;total\;Toxoplasma} \end{array}$$

### Correlative light electron microscopy

Mattek glass-bottom coverslip dishes (P35G-1.5-14-C-GRID) were used to seed and infect HFF-1 cells with transiently transfected parasites that express pTUB-YFP-FAPP1PH-Myc or pTUB-YFP-SidM-P4M-Myc. At 24 hours post inoculation coverslips were fixed with 4% PFA and 0.025% glutaraldehyde (GA) in PBS for 15 min and washed 3X with PBS with a 5 min incubation per wash. Parasites were permeabilized with 0.2% Triton-X (v/v) in PBS for 4 min, washed 1X with PB (0.1 M phosphate buffer pH 7.2), incubated with primary mouse anti-GRA3 antibody secondary antibody directed against Alexa fluor 594 (A11032) (see S2 Table for used dilutions), stained with Hoechst solution (1:5000) for 5 min, and washed 3X with PB with a 10 min incubation per wash. Intracellular parasites were identified using a confocal NikonA1R microscope outfitted with a CFI Plan Apo lambda 60x/1.4 oil objective (to acquire z-sections of parasites with a LAP body to create a map of the parasite location), and a CFI Plan Apo lambda 20x/0.75 objective (to identify the position of parasites with a LAP body within the coordinate system in the glass-gridded coverslip).

Coordinates of interest in the grid were marked with nail varnish on the base of the coverslip. Cells were fixed again with 4% PFA and 0.5% GA in PB overnight at 4 ºC, washed 3X with distilled water (10 min each), post-stained with 1% osmium (OsO_4_) for 15 min and with filtered 2% uranyl acetate for 20 min (water washes between each step – 3X and 10 min incubation each). The samples were dehydrated in a graded ethanol series (30%, 50%, 70%, 80%, 90%, 96%, 100%, 100%, 100%) with incubations of 10 min at each step. Cells on coverslips were embedded in a modified Quetol/Spurr’s resin mixed 1:1 with ethanol for 2 hours, followed by resin alone for 4 hours. To limit embedding to the targeted region of the glass-gridded coverslip, a plastic capsule (both ends open) was placed up-side-down on the coverslip over the varnish mark and incubated overnight at 60°C [81]. Immobilized capsules were filled with resin and incubated at 60°C for 48 hours. Resin blocks were detached by heating the coverslip with a passing flame for 7 sec under the bottom of the coverslip [82]. Serial ultrathin sections (~100 nm) were collected using a Leica UC7 ultramicrotome and mounted on formvar slot grids. Sections were stained with lead citrate for 7–10 min and imaged using an FEI Morgagni 268 transmission electron microscope at 70 KeV with a MegaView III CCD camera and iTEM image acquisition software. Image analyses were performed with ImageJ. Data were collected from eight parasites per experimental condition.

### Statistical analyses

Data obtained from at least three independent biological replicates were presented as mean ± standard error of the mean (SEM). Two-way ANOVA followed by Dunnett’s multiple comparison test was used to compare two or more experimental groups to a single control group. One-way ANOVA followed by Tukey’s multiple comparison test was used to compare two or more experimental groups to every other group. T-test (Mann-Whitney test) was performed to compare the means of two groups. Data were calculated and illustrated using GraphPad Prism 6 software. A P value < 0.05 was set as threshold for statistical significance.

## Supporting information

S1 Fig Properties of PtdIns4P biosensors used in this study. **(A)** GOLPH3, FAPP1PH are recruited only to the Golgi membrane (green) [83,84], whereas SidM-P4M localizes to the Golgi, late endosomes (LE) and plasma membrane (PM) [40]. FAPP1PH and SidM-P4M exhibit higher affinity for PtdIns4P pools, with dissociation constants (*K_D_*) in the nanomolar range Among these, SidM-P4M has the highest affinity, enabling binding to membranes with lower PtdIns4P levels. In contrast, FAPP1PH requires an additional protein–protein interaction with Arf1 for recruitment to PtdIns4P-enriched membranes [38]. **(B)** High-affinity PtdIns4P biosensors can be used to sequester PtdIns4P pools in eukaryotic systems. These biosensors compete with endogenous PtdIns4P effectors. When their cellular concentration approaches or exceeds their *K_D_*, sequestration of free PtdIns4P may occur, leading to displacement of endogenous PtdIns4P-binding proteins and disruption of PtdIns4P-dependent cellular functions. This dominant-negative effect is frequently observed upon expression of high-affinity domains due to their low *K_D_* values [85]. Previous studies have stablished that expression of FAPP1PH [86,87] and SidM-P4M [39] can be used as a effective tools to inhibit and study PtdIns4P signaling in plant and mammalian cells. Created in BioRender. https://BioRender.com/06jf4im.
(TIF)

S2 Fig Expression of PI(4,5)P_2_ and PI(3,4)P_2_ biosensors does not induce LAP body formation. **(A)** Widefield fluorescence microscopy of live intracellular parasites co-expressing the indicated PIP biosensors: FAPP1PH-RFP and 2xPHPLCδ-EGFP or FAPP1PH-RFP and YFP-TAPP1PH. The distribution of neither PIP_2_ biosensors was affected by PtdIns4P pool sequestration at the Golgi-TGN and post-TGN compartments. **(B)** Widefield fluorescence microscopy of live intracellular parasites expressing the indicated PIP biosensors, classified by the DG reporter distribution phenotype: DG-less, normal (arrows) or LAP body (arrow heads). Expression of 2xPHPLCδ-EGFP or YFP-TAPP1PH in reporter strains RH-GRA3-RFP or RH-GRA2-RFP did not result in LAP body formation. **(C-D)** Quantification of PV phenotypes as reported by **(C)** GRA3-RFP or **(D)** GRA2-RFP cargo (DG-less, normal, LAP body) as a function of co-expression with the indicated biosensor (n = 50 PVs). Data are represented as mean ± SEM of three independent biological replicates. Statistical analysis compared the control reporter strain (RH-GRA3-RFP or RH-GRA2-RFP) with parasites co-expressing the DG reporter and the appropriate PIP biosensor regarding LAP body phenotype. Two-way ANOVA followed by Dunnet’s multiple comparison test was used to determine statistical significance; p > 0.05 (ns); p < 0.05 (*); p < 0.01 (**); p < 0.001 (***); p < 0.00001 (****).
(TIF)

S3 Fig Airyscan z-stacks used for the generation of Z-projections of (A) RH-WT and (B) RH-SidM-P4M parasites in the G1/S stage of the cell cycle and stained with anti-GRA3 immunoglobulin. (TIF)

S4 Fig A 3D reconstruction of the LAP body reveals a network of small GRA3- and PtdIns4P-containing vesicles in parasites during G1/S stage. **(A-B)** Z-projections of RH-WT and RH-SidM-P4M parasites stained with anti-GRA3 antibody. Parasite DNA is visualized using Hoechst (blue). **(A)** (Left) Control RH-WT parasites show DG cargo (GRA3) distributed in discrete puncta throughout the cytoplasm and PVM. The G1/S phase was identified by the presence of a single undivided apicoplast in the cells of interest (asterisk). (Right) A 3D-Reconstruction show the absence of a LAP body in the subapical area (dashed square). **(B)** (Left) RH-SidM-P4M parasites in the G1/S phase display a LAP body (dashed square) composed of numerous sDGs enriched in GRA3 cargo and PtdIns4P biosensor. (Right) A 3D-Reconstruction of the LAP body confirms spatial co-localization of GRA3 and PtdIns4P reporter signals (indicated by black arrows), While distinct or concentrated PtdIns4P biosensor and GRA3 cargo signal is also observed (indicated by white arrows).
(TIF)

S5 Fig Parasites expressing SidM-mAID grow through 4 passages and maintain LAP bodies. **(A)** Tir1 YFP-SidM-mAID Clone G10 parasites were treated with or without IAA for 7 days. Parasites were fixed, stained for GRA3, counterstained with DAPI, and imaged. Scale bar = 10µm. **(B)** Quantification of PVs binned according to GRA3 phenotype (DG-less, normal or LAP body) in clone G10 and C4 (n = 100 PVs each clone) parasites treated with or without IAA for 7 days. Data show the mean ± SEM of three independent experiments for clone G10 and two independent experiments for clone C4. Statistical analyses compared the LAP body phenotypes between – IAA (parasite expressing SidM-P4M-YFP-mAID) and +IAA conditions from the same clone. Statistical significance was calculated using two-way ANOVA followed by Šídák multiple comparison test; p > 0.05 (ns); p < 0.05 (*); p < 0.01 (**); p < 0.001 (***); p < 0.00001 (****).
(TIF)

S6 Fig Airyscan z-stacks used for the generation of Z-projections of (A) RH-WT and (B) RH-SidM-P4M parasites undergoing mitosis and stained with anti-GRA3 immunoglobulin. (TIF)

S1 Video RH-WT parasites in the G1/S phase, stained with a GRA3 antibody. The fluorescent signal corresponding to DGs (magenta) is shown.
(MP4)

S2 Video RH-WT parasites in the G1/S phase, stained with a GRA3 antibody. A 3D reconstruction of the fluorescent signal corresponding to DGs (magenta) is shown.
(MP4)

S3 Video RH-SidM-P4M parasites in the G1/S phase, stained with a GRA3 antibody. The fluorescent signal corresponding to sDGs (magenta) and PtdIns4P-positive vesicles (green) are shown.
(MP4)

S4 Video RH-SidM-P4M parasites in the G1/S phase, stained with a GRA3 antibody. A 3D reconstruction of the fluorescent signal corresponding to sDGs (magenta) and PtdIns4P-positive vesicles (green) are shown.
(MP4)

S5 Video RH-SidM-P4M parasite during division, stained with a GRA3 antibody. The fluorescent signal corresponding to sDGs (magenta) and PtdIns4P-positive vesicles (green) are shown.
(MP4)

S6 Video RH-SidM-P4M parasite during division, stained with a GRA3 antibody. A 3D reconstruction of the fluorescent signal corresponding to sDGs (magenta) and PtdIns4P-positive vesicles (green) are shown.
(MP4)

S1 Table Primers used for subcloning *T. gondii* intracellular organelle marker genes used into the pTub expression vector. (DOCX)

S2 Table List of antibodies. (DOCX)
