# Supplementary material for: Phosphatidylinositol-4-phosphate signaling regulates dense granule biogenesis and maturation in Toxoplasma gondii
Source: PLoS Pathog. 2026 Jul 24;22(7):e1014451. doi: 10.1371/journal.ppat.1014451 (PMC13472501; doi:10.1371/journal.ppat.1014451)
Supplement: Supplementary file 1 [file ppat.1014451.s013.docx]

**S1 Table: Primers used for subcloning *T. gondii* intracellular organelle marker genes used into the pTub expression vector.**

| **Construct** | **Primer** |
| --- | --- |
| Myc-GRA3-RFP | F: 5’-CGC AGATCTATGGACCGTACCATATG-3’  R: 5’-AGACCTAGGTTTCTTGGAGGCTTTGTC-3’ |
| Myc-GRA2-RFP | F: 5’-CGCAGATCTATGTTCGCCGTAAAAC-3’  R: 5’-ATACCTAGGCTGCGAAAAGTCTGGGAC-3’ |
| Myc-Rop1-RFP | F: 5’-GGAAGATCTAAAATGGAGCAAAGGCTGCCA-3’  R: 5’-GTTCCTAGGAAATTGCGATCCATCATCCTG-3 |
| Myc-IMC1-RFP | F: 5’- AAACCTAGGATGGCCTCCTCCGAGGACG-3’  R: 5’-AAAGCGGCCGCTTAGGCGCCGGTGGAGTGGCGG-3’ |
| Myc-2xPHPLCδ-EGFP | F: 5’-GCGAGATCTGATATACATATGGACTC-3’  R: 5’-AAACCTAGGGCTCACCATGGT-3’ |
| YFP-FAPP1PH-Myc | F: 5’-GAAGATCTAAAATGGAGGGGGTGTTGTACAAG-3’  R: 5’- GACCTAGGGTCCTTGTATCAGTCAAACATGC-3’ |
| YFP-SidM-P4M-Myc | F: 5’-AAAAGATC AAGTCCGGACTCAGATC-3’  R: 5’-AAACCTAGGTTTTATCTTAATGGTTTG-3’ |
| SidM-P4M-YFP-mAID | F: 5’-aaacctaggtccggactcagatccacg-3’  R: 5’-cccgctagcttttatcttaatggtttg-3’ |
| YFP-GOLPH3-Myc | F: 5’-AAAAGATCTATGACCTCGCTGACCCAG-3’  R: 5’- AAACCTAGGTTACTTGGTGAACGC-3’ |
| YFP- FAPP1PH (K7A/R18L)-Myc | R18L:  F: 5’-CTCACAGGCTGGCAGCCTCTTTGGTTTGTTTTAGATAATGG-3’  R: 5’-CCATTATCTAAAACAAACCAAAGAGGCTGCCAGCCTGTGAG-3’  K7A:  F: 5’-GGAGGGGGTGTTGTACGCGTGGACCAACTATCTCAC-3’  R: 5’- GTGAGATAGTTGGTCCACGCGTACAACACCCCCTCC-3’ |
| YFP-TAPP1PH-Myc | F: 5’-GAAGATCTAAAATGTTTACTCCTAAACCACCTCAAGATAG-3’  R: 5’-GACCTAGGATGCTCAGAAGATGCTGATCTGC-3’ |
| Myc-Rab5.1 | F: 5’-GGATCCATGAGAGGTTTCGAATCTGC-3’  R: 5’-GCGGCCGCTCAACTTTTGCCTCCACATG-3’ |
