# Supplementary material for: Phosphatidylinositol-4-phosphate signaling regulates dense granule biogenesis and maturation in Toxoplasma gondii
Source: PLoS Pathog. 2026 Jul 24;22(7):e1014451. doi: 10.1371/journal.ppat.1014451 (PMC13472501; doi:10.1371/journal.ppat.1014451)
Supplement: Supplementary file 2 [file ppat.1014451.s014.docx]

**S2 Table: List of antibodies.**

| **Antibody** | **Dilution** | **Source/Brand** |
| --- | --- | --- |
| Mouse anti-GRA3 | 1:500 | BEI Resources, NIAID, NIH: Monoclonal Anti-Toxoplasma gondii Dense Granule Antigen 3, Clone T6 2H11 (produced in vitro), NR-50269 |
| Mouse anti-IMC | 1:500 | Dr. Gary Ward |
| Rat anti-TgSORTLR | 1:250 | Dr. Stanislas Tomavo |
| Mouse anti-MIC3 | 1:500 | Dr. Jean François Dubremetz |
| Rabbit anti-Myc tag | 1:400 | Cell signaling 2272 |
| Anti-mouse Alexa Fluor 594 | 1:500 | Invitrogen A11032 |
| Anti-rat Alexa Fluor 568 | 1:500 | Invitrogen A11077 |
| Anti-mouse Alexa Fluor 568 | 1:1000 | Invitrogen A11004 |
| Anti-rabbit Alexa Fluor 647 | 1:500 | Invitrogen A21245 |
| Mouse anti-RON9 | 1:200 | BEI Resources, NIAID, NIH: Monoclonal Anti-*Toxoplasma gondii* Rhoptry Neck Protein RON9, Clone T5 2A7 (produced *in vitro*), NR-50263 |
